# What Makes Ribosome-Mediated Transcriptional Attenuation Sensitive to Amino Acid Limitation?

**DOI:** 10.1371/journal.pcbi.0010002

**Published:** 2005-06-24

**Authors:** Johan Elf, Måns Ehrenberg

**Affiliations:** Department of Cell and Molecular Biology, Uppsala University, Uppsala, Sweden; University of Queensland, Australia

## Abstract

Ribosome-mediated transcriptional attenuation mechanisms are commonly used to control amino acid biosynthetic operons in bacteria. The mRNA leader of such an operon contains an open reading frame with “regulatory” codons, cognate to the amino acid that is synthesized by the enzymes encoded by the operon. When the amino acid is in short supply, translation of the regulatory codons is slow, which allows transcription to continue into the structural genes of the operon. When amino acid supply is in excess, translation of regulatory codons is rapid, which leads to termination of transcription. We use a discrete master equation approach to formulate a probabilistic model for the positioning of the RNA polymerase and the ribosome in the attenuator leader sequence. The model describes how the current rate of amino acid supply compared to the demand in protein synthesis (signal) determines the expression of the amino acid biosynthetic operon (response). The focus of our analysis is on the sensitivity of operon expression to a change in the amino acid supply. We show that attenuation of transcription can be hyper-sensitive for two main reasons. The first is that its response depends on the outcome of a race between two multi-step mechanisms with synchronized starts: transcription of the leader of the operon, and translation of its regulatory codons. The relative change in the probability that transcription is aborted (attenuated) can therefore be much larger than the relative change in the time it takes for the ribosome to read a regulatory codon. The second is that the general usage frequencies of codons of the type used in attenuation control are small. A small percentage decrease in the rate of supply of the controlled amino acid can therefore lead to a much larger percentage decrease in the rate of reading a regulatory codon. We show that high sensitivity further requires a particular choice of regulatory codon among several synonymous codons for the same amino acid. We demonstrate the importance of a high fraction of regulatory codons in the control region. Finally, our integrated model explains how differences in leader sequence design of the *trp* and *his* operons of *Escherichia coli* and *Salmonella typhimurium* lead to high basal expression and low sensitivity in the former case, and to large dynamic range and high sensitivity in the latter. The model clarifies how mechanistic and systems biological aspects of the attenuation mechanism contribute to its overall sensitivity. It also explains structural differences between the leader sequences of the *trp* and *his* operons in terms of their different functions.

## Synopsis

When cells grow and divide, they must continually construct new proteins from the 20 amino acid building blocks according to the instructions of the genetic code. Proteins are made by large macromolecular complexes, ribosomes, where information encoded as base triplets (codons) in messenger RNA sequences, transcribed from the DNA sequences of the genes, is translated into amino acid sequences that determine the functions of all proteins. Rapid growth of cells requires that the supply of each free amino acid is balanced to the demand for it in protein synthesis.

The present work mathematically models a common control mechanism in bacteria, which regulates synthesis of amino acids to eliminate deviations from balanced supply and demand. The mechanism “measures” the speed by which the ribosome translates the codons of a regulated amino acid. When supply is less than demand, translation of these “control” codons is slow, which is sensed by the mechanism and used to increase synthesis of the amino acid.

This paper explains why the mechanism is “hyper-sensitive” to relative changes in supply and demand, and why it is differently designed for control of the enzymes that synthesize the amino acids histidine and tryptophan.

## Introduction

Ribosome-mediated attenuation of transcription [1] is commonly used for control of expression of amino acid biosynthetic operons in bacteria [2]. There are several other types of attenuation mechanisms [3], but “attenuation” in this paper specifically refers to its ribosome-mediated variant. By this mechanism, the fate of an initiated round of transcription depends on the outcome of a race between the RNA polymerase (RNAP), transcribing the leader of the regulated operon, and a ribosome, translating the leader transcript. The open reading frame in the leader contains two or more regulatory codons cognate to the amino acid that is synthesized by the enzymes encoded by the mRNA of the operon [1]. If the supply of the amino acid is insufficient to meet the demand from protein synthesis, the ribosome will be slowed down on these codons and transcription will continue into the structural genes. If, in contrast, the amino acid supply is in excess, the ribosome will move quickly over the regulatory codons, which results in the formation of an RNA hairpin that signals termination (attenuation) of transcription. Ribosome-mediated transcriptional attenuation was first found in the *trp* operon of *Escherichia coli* [1,4] and the *his* operon of *Salmonella enterica* serovar Typhimurium *(Salmonella typhimurium)* [5,6]. Attenuation mechanisms have since been identified for the *leu, thr, ilvGMEDA, ilvBN,* and* pheA* operons of *E. coli* and* S.* *typhimurium* [2], as well as for biosynthetic operons in many other organisms [7].

Attenuation control mechanisms up-regulate operon expression only in response to a reduced speed of translation of regulatory codons, which has led to the idea that these control mechanisms must reduce the rate of growth of bacteria. The reason is that amino acid production will start to increase only when the rate of peptide elongation and, therefore, the current growth rate have already fallen below their maximal values. This is in contrast to repressor systems controlled by amino acid pools, which can regulate gene expression without reduction of the rate of protein elongation [8]. In their book [9], Ingraham, Maaløe, and Neidhardt describe this as a paradox, and suggest that attenuation must be very sensitive, so that the rate of protein synthesis needs to be slowed down only marginally to activate expression of attenuation-controlled operons. In the present study we show that, indeed, attenuation of transcription can be truly hyper-sensitive in accordance with their expectation.

**Figure 1:**
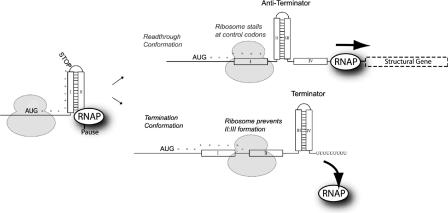
The Leader-Transcript of the *trp* Operon in *E. coli* Attenuated transcription results in a 141-nucleotide (nt) transcript. Aborted transcription of the paused RNAP results in a 91-nt transcript. The transcript includes an open reading frame of 15 codons, encoding a very short-lived 14-residue leader peptide. The RNAP is released from the pause site when the seventh codon is read [17]. Two of the three codons in the control region (10 and 11) are *trp* codons. Also, ribosome stalling on the *arg*-codon (12) prevents *I:II*-hairpin formation and attenuation (see Figure 6). After reaching the stop codon, the ribosome dissociates in about 1 s. The *II:III* and *III:IV* conformations have similar stabilities, and the terminator is formed with 50% probability when the ribosome reaches the stop codon and dissociates from the transcript before region *IV* is available. This determines the basal read-through level of 10–15%.

Our starting point is a consensus scheme for attenuation of transcription (Figure 1), and we model mathematically how gene expression responds to amino acid limitation. We take into account the observation that the RNAP and the ribosome start their race over the leader transcript in synchrony [10–12]. This timing, which we show is essential for the sensitivity of attenuation, was not considered in early models of attenuation [13–15] and was first introduced in a comparative study of repressor and attenuation control of amino acid biosynthetic operons [8]. There are two main sources for hyper-sensitivity of attenuation [8]: one related to the multi-step character of the ribosome and the RNAP movements along the operon leader, and the other to the frequency of the amino-acid-starved codons in the mRNAs on all ribosomes of the cell. The former is a property of the mechanism per se, and the second is the property of the mechanism in the context of a growing cell. Here, we will clarify and refine the model by including how the selective charging of tRNA isoacceptors [16] affects the sensitivity of attenuation. We will also extend the model to include mixed codon usage in the attenuation control region as well as modulation of the basal expression level through ribosomal release at the stop codon. These more detailed aspects of the attenuation mechanism turn out to be necessary to explain the striking difference in design of the *trp* and *his* leaders in both *E. coli* and *S. typhimurium*.

A scheme for attenuation control of the *trp* operon, mainly based on the experimental work by Yanofsky and co-workers [2,17], is shown in Figure 1. The leader sequence contains, starting from the 5′ end, the initiation codon (AUG) for mRNA translation followed by region *I*, in which there are *m* regulatory codons for the amino acid that is synthesized by the enzymes encoded by the controlled operon. Region *I* is followed by region *II* and then by a strong pause site for the RNAP. Further downstream there are *n* RNA bases, including leader regions *III* and *IV*. Mutually exclusive hairpin structures can be formed by regions *II:III* or *III:IV*.

When the RNAP has reached the pause site, it stops and remains there until a ribosome starts melting the hairpin structure formed by regions *I* and *II* [10–12]. Then the RNAP resumes transcription in synchrony with the movement of the ribosome. If the ribosome is slow in translating regulatory codons in the control region *I*, it will remain there when the RNAP finishes transcription of region *IV* (Figure 1). In this case, the *II:III,* but not the *III:IV,* hairpin is formed and the RNAP will continue into the open reading frames of the structural genes. When, in contrast, the amino acid synthetic activity of the enzymes encoded by the operon supersedes demand, the ribosome will move quickly over the regulatory codons in region *I,* which prevents formation of the anti-terminator loop *II:III*. In this case, the hairpin *III:IV* will be formed when the RNAP finishes transcribing region *IV,* which leads to termination of transcription. If the ribosome reaches the stop codon and dissociates from the leader RNA before hairpin *III:IV* is formed but after hairpin *II:III* can be formed, then termination may be aborted in spite of rapid translation of regulatory codons. The probability of this event determines the basal expression of the* trp* attenuator [18].

Under conditions when initiation of leader peptide synthesis is shut down, the RNAP eventually dissociates spontaneously from its pausing state and continues transcription. In this case, the leader transcript preferentially forms the *I:II* and the *III:IV* termination structure [17]. This phenomenon, known as super attenuation, is not an integrated part of our model, but one of its consequences will be discussed.

## Results

### Mathematical Modeling of Attenuation of Transcription

Molecular details from extensive experimental work will here be used to analyze the sensitivity of ribosome-dependent attenuation of transcription in growing cells of *E. coli* and *S. typhimurium*.

Let *R(t)* be the probability that the ribosome at time *t* is in the control region of the mRNA leader with its *m* regulatory codons (Figure 1). At time zero, the RNAP resumes transcription from its pausing state by the approach of a ribosome, so that RNAP and ribosome take off in synchrony from well-defined positions on the leader. Let *f(t)* be the probability density for the time *t* at which RNAP leaves the *n*th base, counted from the pause site (Figure 1). The probability *Q,* that initiation of transcription of the leader of the operon is continued into its structural genes**,** is the probability *R(t),* that the ribosome remains in the control region at any time *t* when the RNAP moves from the *n*th base with probability density *f(t),* i.e.


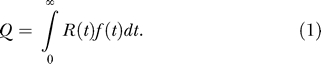


To simplify, we assume that each one of the *m* codons in the control region is translated with the first-order rate constant *k,* which depends on the rate of supply of the controlled amino acid compared to ribosomal demand. Then, the movement of the ribosome is defined by a Poisson Process [19], where the probability Po(*i,kt*) that the ribosome is at codon* i* at time* t* is given by (kt)^*i*^ *e^*−kt*^/i*!. Accordingly, the probability *R(t)* is


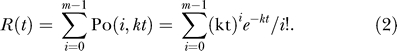


The first stochastic treatment of ribosome movement during mRNA translation was introduced by von Heijne et al. almost 30 years ago [20]. They discussed possible effects of mRNA secondary structures on ribosomal step times, and suggested that hairpin formations could slow down the movement of the ribosome. Direct measurements of ribosomal step times in *E. coli* revealed, however, that the rate of codon translation is only marginally affected by stable secondary structures in the open reading frames of messenger RNAs [21]. This suggests rapid melting of the *I:II* hairpin in the attenuation leader, motivating our assumption of unhindered ribosome movement during translation of this region.

To simplify further, we also assume that each one of the *n* bases downstream from the pause site is transcribed with the same first-order rate constant *q,* so that the movement of the RNAP is also a Poisson Process. Then, the probability density *f(t)* for the time *t* when the RNAP leaves base *n* is given by


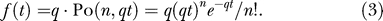


This model is apparently similar to Manabe's [13], but there is an important difference. Here, we have accounted for the experimentally identified pause site for the RNAP, which synchronizes the movements of RNAP and ribosome [10–12]. The pause site was not known when Manabe's model was formulated, but is included in later models of attenuation [14,15]. These, however, miss the point, that the RNAP is actively released by the ribosome-dependent melting of the hairpin structure that defines the pause site (Figure 1). This synchronization of translation and transcription [10–12] is in fact a strict requirement for a hyper-sensitive attenuation mechanism [8].

The probability for continued transcription into the structural genes by early ribosome termination at the stop codon in the open reading frame of the leader sequence [18] is described below, and its effects will be discussed when we compare the *trp* and *his* attenuator mechanisms.

### Partitioning of Hyper-Sensitivity into a Mechanistic and a System-Dependent Factor

The signal *s* for attenuation of transcription of amino acid biosynthetic operons is the rate of amino acid supply normalized to ribosomal demand [22]. The response is the probability *Q* in Equation 1, that initiation of transcription of the leader leads to expression of the structural genes of the operon. Steady-state sensitivity is defined as the logarithmic gain or, equivalently, the sensitivity amplification *a*_Qs_ [23,24], i.e., the relative change in *Q* normalized to a relative change in *s*:


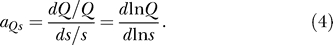


The sensitivity *a*_Qs_ can be partitioned into the factors *a_Qk_* and *a_ks_*. The first is the relative change in *Q* caused by a relative change in the rate *k* of translation of a regulatory codon in the open reading frame of the leader. The second is the relative change in *k* caused by a relative change in the signal *s,* i.e.


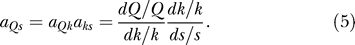


The first factor* a_Qk_* depends on the attenuation mechanism per se, and the second factor *a_ks_* is a system parameter, which relates the rate of reading of a starved codon to the rate of supply of its cognate amino acid in a growing bacterial cell.

### Hyper-Sensitivity by Competing Multi-Step Processes

To clarify the origin of hyper-sensitivity relating to *a_Qk_* in Equation 5, we consider first an attenuation-like mechanism, where there is only one regulatory codon (*m* = 1), translated with rate constant *k*′, in the open reading frame of the mRNA leader and only one rate-limiting step (*n* = 1) with rate constant *q*′ for the RNAP. Then, *Q* in Equation 1 is given by the hyperbolic relation [19]


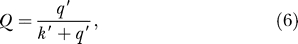


and *a_Qk′_* by


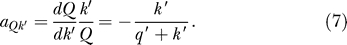


*a_Qk′_* asymptotically approaches its largest absolute value, one, when *k*′ increases beyond limit and *Q* goes to zero.

Next, consider the case when the RNAP transcribes many bases in the leader sequence (*n* >> 1), while the ribosome translates a single, rate-limiting regulatory codon (*m* = 1). With *q = nq′*, the probability *Q* is


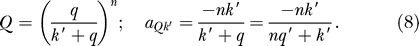


**Figure 2:**
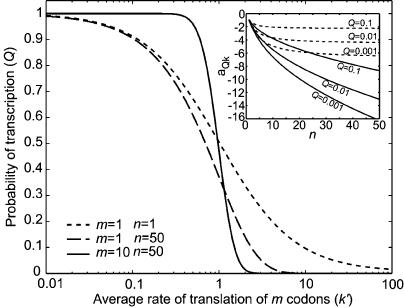
The Probability *Q* of Read-Through of the Attenuation Leader Is Plotted as a Function of *k′ =* 1*/τ* Where *τ* Is the Average Time for Translation of All the *m* Codons The translation rates of the individual codons are *k = k′ · m*. The average time to transcribe the *n* nucleotides is kept constant at 1/*q′ =* 1*s*. The transcription rate for an individual nucleotide is *q = q′ · n*. For *m* = 1, *n* = 1 the response function is given by Equation 6; for *m* = 1, *n* = 50 the response function is given by Equation 8. These curves should be compared to the much more sensitive response that is reached with two competing multi-step processes, as in attenuation (e.g., *m* = 10, *n* = 50). *Insert:* The sensitivity amplification a_Qk_ is plotted as a function of *n,* while *k* is kept fixed, and *q* is changed to get the read-through probabilities *(Q)* corresponding to the different curves. The two curve families correspond to the ordinary multi-step mechanism with *m* = 1 (dashed) and the competing multi-step mechanisms with *m = n* (solid), respectively. The dashed curves approach ln(*Q*) as n →∞, whereas the solid curves all go to −∞ for all values of *Q*.

Here, *a_Qk_* asymptotically approaches its largest absolute value, *n,* when *k*′ increases indefinitely and *Q* goes to zero, as in the previous case. In the limit that *n* → ∞, *Q = e^−k′/q′^* and *a_Qk'_ = −k′/q′* (see insert in Figure 2). Accordingly, when a single-step process competes with a multi-step process, the sensitivity amplification can be numerically very large, at the cost of a small *Q* [25].

In realistic attenuation mechanisms, however, both *m* and *n* are larger than one, as exemplified by the *E. coli* case, where *n* = 50–100 and *m* = 2–16 [2]. When *m* increases, the relative uncertainty (standard deviation normalized to mean) of the time the ribosome spends in the control region decreases. Also, when the time that the RNAP leaves base *n* is well defined, the time during which *R(t)* is averaged in Equation 1 is short. Accordingly, when *R* makes a sharp transition from zero to one, the absolute value of the sensitivity amplification can be very high, even for *Q* values close to one (Figure 2). In the interesting case when *m,n* → ∞, *Q* is a step function with infinite sensitivity to changes in *k* for all values of *Q* at the point of operation of the control mechanism.

### Hyper-Sensitivity in Attenuation due to Low Frequency of Regulatory Codons in Bulk mRNA

To understand the origin of hyper-sensitivity relating to *a_ks_* in Equation 5, one must take into account that the steady-state rate *j_aa_* of amino acid supply from the amino acid biosynthetic enzymes must equal the steady-state rate *f*[*R*]*v* of its consumption [8]. In the case when there is one codon per amino acid, *f* is its frequency of occurrence on all translating ribosomes in the cell, [*R*] is the ribosome concentration, and *v* is the average rate of protein elongation per ribosome. The rate *v* is the inverse of the average time to translate individual codons on translating ribosomes, weighted by their usage frequencies [16]. To simplify, we take 1/*k* to be the average time to translate a particular regulatory codon and 1/*k*_max_ to be the uniform time to translate all other codons [22], which gives the flow balance relation [8]


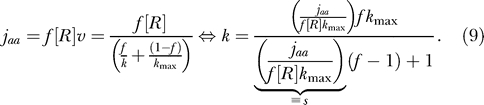


The signal* s* is in Equation 9 is *j_aa_* divided by the maximally possible rate, *f*[*R*]*k_max_,* of consumption of the controlled amino acid when it is supplied in excess. Accordingly, *s* is zero when amino acid supply is shut down, and *s* is one at saturating supply rate. The sensitivity parameter *a_ks_* in Equation 5 follows by differentiating *k* in Equation 9 with respect to *s* and multiplying the derivative by *s/k:*


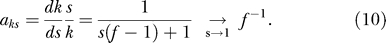


**Figure 3:**
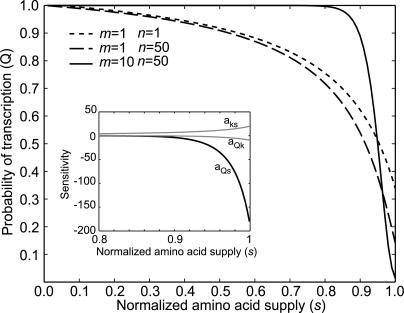
The Probability *Q* of Read-Through of the Leader Is Plotted as a Function of the Signal *s* For the curve with *m* = 10, *k* is given by Equation 9, and for the curves with *m* = 1, *k* is one-tenth of this value. *q′ =* 1*s* ^−1^ for all curves. The usage frequency of the amino acid in proteins is *f* = 1/20, and the maximal rate of codon translation at full supply of amino acid is *k_max_*= 20*s* ^−1^. *Insert:* The contributions of the two factors to the sensitivity a_Qs_= a_Qk_a_ks_ is illustrated for the case with *m* = 10 and *n* = 50. a_ks_ approaches 1/*f* as *s* → 1. The asymptotic behavior of a_Qk_ is described in Figure 2.

Because *f* is small [0.001–0.1], *a_ks_* contributes with a factor in the interval 10–1,000 to the overall sensitivity amplification *a_Qs_* in Equation 5 for values of the signal *s* close to and below one. This remarkable result can be explained as follows. When one amino acid is rate-limiting for protein synthesis, the total rate of peptide elongation in the cell is determined by its rate of supply [8,22]. When *s* is close to one and the usage frequency *f* of the regulatory codon is small, a given reduction in total rate of protein synthesis corresponds to the decreased rate of translation of only a small fraction of the currently translated codons. Hence, a relative decrease in an *s* value close to one leads to a 1*/f* times larger relative reduction in the rate of reading of the starved codon. When *s* decreases, an increasing fraction of ribosomes in the cell will be programmed with the starved codon, so that the current *f* value increases from a small value toward one, at which point the effect vanishes (Figure 3).

### Selective Charging of tRNA Isoacceptors and the Sensitivity of Attenuation of Transcription

So far, we have neglected that the genetic code is redundant [26], in that there often are several codons cognate to one amino acid that are translated by several isoaccepting tRNAs [27]. There exists a strong bias among the codons in attenuation leaders [2], and it was suggested early that this bias has evolved to maximize the sensitivity of attenuation mechanisms by the choice of rare codons in attenuation leaders, with the* leu* operon as the paradigmatic example [2]. This explanation must, however, be an oversimplification, since the bias in other attenuation leaders favors major (e.g., as with the *thr* operon with eight ACC codons) or intermediate codons [16].

The mystery of biased usage of regulatory codons in attenuation leaders in *E. coli* was recently resolved by the theory of selective charging of isoaccepting tRNAs during amino acid starvation [16]. This theory, fed with experimental data on total tRNA concentrations and codon usage on translating ribosomes [28,29], was used to identify those regulatory codons in attenuation leaders in *E. coli* for which the rate of translation is most sensitive to variation in the rate of supply of their cognate amino acid. In each case, the experimentally observed codon bias [2] favors the regulatory codon for which the translation rate is most sensitive to amino acid limitation [16].

Since then, selective charging in Thr, Leu, and Arg tRNA isoacceptor families has been verified experimentally [30] and found to be qualitatively in accordance with theory [16]. The theory has also successfully predicted how ribosomal by-passing [31] responds to inhibition of the seryl-tRNA synthetase, when the ribosomal A site has a codon read by either a Ser3 or a Ser5 isoacceptor [32].

The theory of selective charging assumes that the rate by which a charged tRNA isoacceptor is deacylated is in proportion to the frequency by which its cognate codons occur on translating ribosomes, and that the rate of aminoacylation of an isoacceptor is in proportion to the concentration of its deacylated form. From this follows that an isoacceptor with high total concentration and low codon usage will remain charged with amino acid, while an isoacceptor from the same isoacceptor family with low total concentration and high codon usage will lose charging when the supply of their common amino becomes limiting for protein synthesis [16].

**Figure 4:**
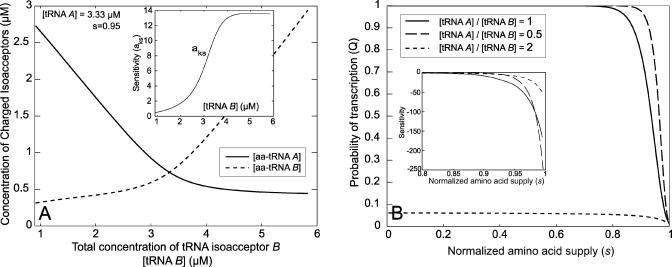
The Choice among Synonymous Codon (A) The predicted concentration of aminoacylated tRNA isoacceptor *A* (solid) and *B* (dashed) is plotted as a function of the total concentration of isoacceptor *B* for a fixed but limiting supply of the cognate amino acid (*s* = 0.95). The codon usage of *a* and *b* are both equal to 2.5%, and the total concentration of tRNA isoacceptor *A* is kept constant at 3.33 μM. *Insert*: The sensitivity in rate of reading *a* codons by the *A* isoacceptor in response to a change in amino acid supply is plotted over the same interval as the main figure. (B) The probability of read-through is plotted as a function of normalized amino acid supply rate. The attenuation control codon *a* is translated by tRNA isoacceptor A. The same amino acid is also encoded by another codon *b,* which is translated by tRNA isoacceptor *B*. The codon usage of *a* and *b* are both equal to 2.5%. The different curves correspond to different ratios between the total concentrations of isoacceptor *A* and *B,* as indicated in the figure. As *m* = 10, *n* = 50, and *k_max_* = 20*s* ^−1^, the situation where the concentrations are equal corresponds to the solid curve in Figure 3. *Insert:* The sensitivity a_Qs_ for the three cases. More details about the calculations are given in the Supporting Information and reference [16].

To exemplify, consider a simple case with two different isoacceptors *(A* and *B),* where *A* reads only codon* a* and *B* reads only codon *b*. Codon *a* occurs in the attenuation leader of the mRNA that encodes the enzymes that synthesize the amino acid cognate to *A* and *B*. When codons *a* and *b* occur on translating ribosomes with the same frequency, the aminoacylated forms of isoacceptors *A* and *B* are consumed by the same rate in protein synthesis. In the steady state, this also means that the deacylated forms of *A* and *B* must be charged with amino acid at identical rates. From this follows further, that when the total concentrations of *A* and *B* in the cell are different, the concentrations of their aminoacylated forms may differ greatly when their cognate amino acid is rate-limiting for protein synthesis. This is illustrated in Figure 4A, which shows the concentrations of the aminoacylated forms of *A* and *B* when the total concentration of *A* is constant and the total concentration of *B* is varied at an *s* value (Equation 4) [22] of 0.95 with equal codon usage frequencies of 2.5% for *a* and *b*. When the total concentration of *B* increases from small values, the concentration of charged *B* goes up, while the concentration of charged *A* goes down (Figure 4A). Therefore, under amino acid limitation, the charged level of isoacceptor *A* depends strongly on the total concentration of isoacceptor *B,* and this dependence is also reflected in the sensitivity of the attenuation mechanism with *a* as regulatory codons (insert in Figure 4A). When the total concentration of *B* is small, the sensitivity of attenuation is very small, but increases sharply toward its asymptote with increasing *B* concentration. At the asymptote, *B* isoacceptors are fully charged, and *b* codons are read with maximal rate. In this limit, the sensitivity follows from Equation 1, with the codon frequency *f* defined by the usage of *a* codons, i.e., 2.5% in this case (Figure 4B).

In more realistic scenarios, several isoacceptors may read the same codon, which leads to more complex kinetics [16], but the same principles apply.

### The Fraction of Regulatory Codons in the Control Region

In realistic attenuation systems, there is normally a mixture of regulatory and other codons in the control region, defined as the mRNA leader region from the position where the RNAP is released up to the last codon where ribosome stalling promotes anti-terminator conformation. The sensitivity of attenuation mechanisms depends on the fraction of regulatory codons in the control region. To see this, we take into account that regulatory and other codons are translated with different rates by using the master equation [33]


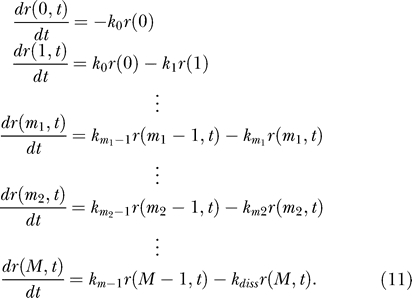


*r(i,t)* is the probability that the ribosome is at codon* i* at time *t*. The enumeration is defined such that the RNAP is released when codon* i* = 0 is translocated into the A site of the ribosome. Codon *m_1_* is the first, and *m_2_* is the last codon where ribosome stalling causes anti-termination and codon *M* is the stop codon. *k_i_* is the rate by which codon *i* is read, and *k_diss_* is the rate by which the ribosome dissociates from the stop codon (see next section). The probability that the ribosome is in the part of the control region that promotes anti-terminator conformation at time *t* is


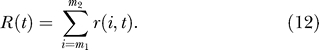


Equation 12 generalizes Equation 2 for *R(t)* and should be used in Equation 1 when there is a mix of different codons in the control region or when ribosome stalling in only a part of the control region can promote anti-terminator conformation. When all *k_i_ = k* and *m*_1_ = 0, *m*_2_ = *m* − 1, then Equation 12 reduces to Equation 2.

**Figure 5:**
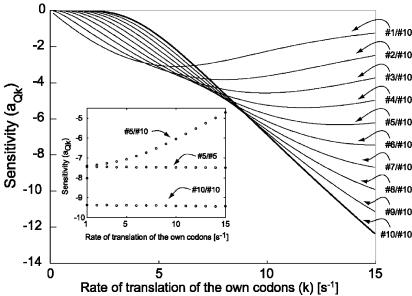
The Sensitivity Amplification a_Qk_ Is Plotted as a Function of the Rate of Translation of Regulatory Codons The different curves correspond to different number of regulatory codons in a control region that has in total ten codons. #*i*/#*j* indicates *i* regulatory codons out of *j* codons in the control region. The rate of translation for non-regulatory codons is 15*s* ^−1^. The RNAP transcribes 50 nt *s* ^−1^ (*q* = 50*s* ^−1^) and the total number of RNAP steps is 80 (*n* = 80). *Insert* **:** The sensitivity amplification a_Qk_ is plotted as in the main figure, but now the rate of transcription *(q)* is scaled such that *Q* = 0.01 for each point.

If the fraction of regulatory codons in the control region is small, the sensitivity of the mechanism will be comparatively small; e.g., if m' codons out of m are regulatory, the sensitivity will be smaller than for a mechanism with m' regulatory codons out of m' codons in the control region. Figure 5 shows how the sensitivity of the mechanism changes when the fraction of regulatory codons decreases from 10 out of 10, (#10/#10) to #1 out of #10 (#1/#10), while the rate of transcription and the number of RNAP steps are constant. The translation rate of the non-regulatory codons is taken to be 15*s* ^−1^. When the translation rates of regulatory and non-regulatory codons are equal in a control region with *m* codons, the sensitivity is proportional to the fraction of regulatory codons multiplied by the sensitivity that pertains when all *m* codons are regulatory. This is illustrated in Figure 5 at the right-most point of the *x*-axis, where regulatory as well as non-regulatory codons have a translation rate of 15*s* ^−1^. When the translation rates of regulatory and non-regulatory codons differ or when there are different codons in the control region, comparison of different cases with respect to sensitivity requires scaling of the transcriptional step rate *q*. The purpose of the scaling is to equalize the *Q*-values in the different cases for the particular rate (*k*) of regulatory codon-translation at which the sensitivity is computed. With this normalization, it is seen that that the sensitivity is higher when there are five regulatory and no other codons, than when there are five regulatory and five non-regulatory codons in the control region. As the rate of translation of regulatory codons decreases, the absolute value of the sensitivity amplification in the mixed codon case increases until the sensitivities converge (insert in Figure 5).

### The Probability of Anti-Terminator Conformation after Ribosome Release from the Stop Codon

If the ribosome reaches the stop codon and dissociates from the leader RNA before hairpin *III:IV* is formed but after hairpin *II:III* can be formed, then termination may be aborted in spite of rapid translation of regulatory codons. The probability *Q_2_* of this event is small for the *his* attenuator but large for the *trp* attenuator, where it sets the high basal expression level [18]. Let *I* be the time integral of the probability that the RNAP is in a region between bases *n*_1_ and *n*_2_ at time *t* when the ribosome is at the stop codon *M* with probability *r*(*M,t*)*,* from which it dissociates with the first-order rate constant *k_diss_:*


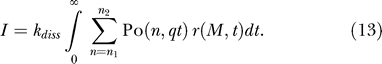


**Figure 6:**
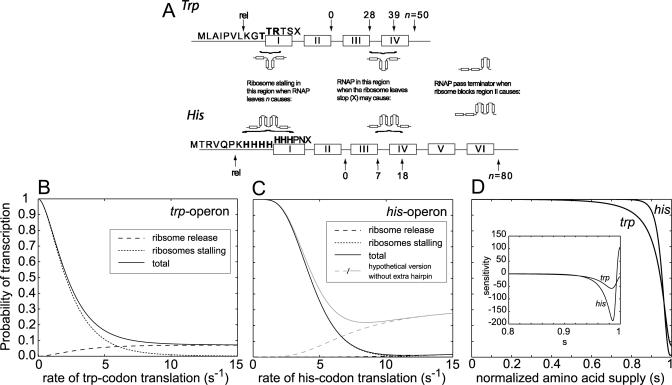
Attenuation Control of the *his* and *trp* Operons (A) The *trp* and *his* attenuation leader regions. For the trp mechanism, codon 3 and 4 after the ribosome has released the RNAP at “rel” are trp codons [2,17]. Ribosome stalling on codon 3, 4, or 5 leads to anti-terminator conformation [42]. If the ribosome releases from the stop codon (codon 8) after segment *III* is available for secondary structure formation (RNAP has transcribed nt 28 + 8) but before more than half of segment *IV* is available (RNAP has transcribed nt 39 + 8), the probability for anti-terminator formation is 50% [18]. The “+ 8” are the bases that have been transcribed but are unavailable for secondary structure formation [43]. For the his mechanism, codon 2 to 8 after the ribosome has released the RNAP are his codons [2,44]. Ribosome stalling on any of these is assumed to lead to anti-terminator conformation. If the ribosome releases from the stop codon (codon 11) after segment *III* is available for secondary structure formation (RNAP has transcribed nt 7 + 8) but before more than half of segment *IV* is available (RNAP has transcribed nt 18 + 8), we assume a 50% chance to get anti-terminator formation. The transcription rate is 50 nt *s* ^−1^ [45], the translation rate of codons other than trp or his is 15*s* ^−1^ [46], and the rate of ribosome release from the stop codon is 1*s* ^−1^. (B,C) The probability of transcription (*y-*axis) past the leader for *trp* and *his* operons as a function of the rate of translation of respective trp and his codons (*x-*axis). The read-through probability is a sum of the probabilities for two mutually excusive events: ribosome stalling on the control codons while the RNAP escapes attenuation (dotted) or ribosome release from the stop codon before the termination hairpin is completed (dashed). In the *trp* case, the second event causes a high basal expression level. (D) The probability of transcription as a function of normalized supply of his and trp, respectively. The codon usage frequency is 1% for trp and 2% for his [28]. k_max_ = 15*s* ^−1^. *Insert*: The sensitivity a_Qs_ over the interval *s* = 0.8 to 1.0. The positive sign of the sensitivity in gene expression for an increase in histidine supply in the narrow range of *s* = 0.995–1.0 is due to reduced probability of ribosome release at the stop codon.

Bases n_1_ and n_2_ define the region where the *II:III* hairpin (Figure 6A) can be formed after dissociation of the ribosome from the stop codon. Accordingly, *n_1_* is the last base in region *III* that can pair with region *II* and *n_2_* as the base in the middle of region *IV.* This is because formation of hairpin *II:III* requires that region *III* is available and that region *IV* has not been completely transcribed. It has been assumed that, when these conditions are fulfilled, formation of either hairpin *I:II* or hairpin *II:III* in the *trp* operon leader will occur with 50% probability, since their stabilities are similar [18]. We will use the same assumption for the *his* attenuator. Accordingly, the probability *Q_2_* is equal to *I*/2, and the total probability *Q_tot_* that the polymerase will continue into the structural genes of the operon is given by *Q+Q_2_,* where *Q* is defined in Equation 1.

### The *trp* and *his* Attenuators

The attenuator mechanisms of the *trp* operon of *E. coli* and the *his* operon of *S.* *typhimurium* have been extensively studied (see [2,34] and references therein).

The* trp* operon is under dual transcriptional control by a Trp-sensitive repressor [35] and an attenuator [36]. During balanced growth, the operon is repressor controlled with a dynamic range of about 70 [37]. During severe Trp starvation, attenuation is turned off, resulting in an additional 10-fold increase in operon expression [37,38]. The dual control of the *trp* operon requires a high basal read-through of the attenuator at excess supply of Trp. This is achieved at a level of 10%–15%, due mainly to termination of translation and ribosome release from the stop codon, before region *IV* has become available to form the terminator structure with region *III* ([18]; Figure 6). Our model predicts a basal level expression of about 8% (Figure 6), when the *trp* attenuator data (legend in Figure 6) are inserted in Equations 1, 12, and 13. It has, however, been suggested that the basal level also contains a contribution from an intrinsic read-through, not included in our model, of about 3%, as estimated under super attenuation conditions [18]. Taking this additional read-through into account makes our model prediction (8 + 3 = 11%) an even better estimate of the experimental estimate (10%–15%).

The *his* operon in *S. typhimurium* **,** in contrast, is only attenuation-controlled and the basal level expression at excess supply of His is supposed to be smaller than the basal expression level of the *trp* attenuator [34]. Our model immediately suggests two different strategies for implementation of low basal read-through in attenuation mechanisms. Their common feature is that the decision to form the transcriptional terminator hairpin occurs well before termination of translation and ribosome release from region *II* (see Figure 1). This can be achieved either by placing the stop codon far downstream in region *II,* so that termination of translation is delayed in relation to formation of the transcriptional terminator *III:IV;* or the same effect can be accomplished by rapid formation of a secondary structure that prevents anti-terminator conformation unless the ribosome is in the control region. The latter option seems to be the design principle of the *his* operon. Here, an extra hairpin has evolved between the RNAP pause hairpin and the transcriptional terminator (Figure 6A). This extra hairpin forms rapidly when the RNAP resumes transcription after pausing, and the model suggests that this is to prevent read-through of the *his* attenuator by translation termination and ribosome release. When, in contrast, the extra hairpin structure is removed from the model, the basal level read-through at saturating His supply increases dramatically (Figure 6C). From this, we suggest that the extra hairpin serves to minimize basal read-through of the *his* attenuator to reduce the metabolic cost of His synthesis when His is supplied externally. The *trp* operon expression, in contrast, is turned off by the Trp repressor when there is Trp in the medium, and the proper action of the dual control system of the *trp* operon requires a high basal read-through of the attenuator.

Our models account for experimental observations regarding the *trp* (Figure 6B) and the *his* operon (Figure 6C). It is, for instance, clearly seen that the *trp* attenuator (Figure 6B) requires a much more severe amino acid limitation for full de-repression and has a much a higher basal read-through level than the *his* attenuator (Figure 6C). We have also estimated the sensitivity (*a*_Qs_; see Equation 4) of gene expression to variation in the amino acid supply signal *s* for each one of these attenuators (Figure 6D). The *his* attenuator is much more sensitive due to the higher number and higher fraction of *his* codons in that control region.

## Discussion

We have described two major sources of hyper-sensitivity of ribosome-dependent attenuation of transcription.

The first relates to the odds for selecting the winner of two competing multi-step (Poisson) processes with synchronized starts. One competitor is the RNAP, transcribing the leader of the operon, and the other is the ribosome, translating regulatory codons in that leader. Synchronous starts for transcription and translation in the attenuator are essential for hyper-sensitivity, and synchrony is achieved by the ribosome-dependent melting of the hairpin structure that makes the RNAP pause in the attenuator (see Figure 1). When the number of regulatory codons to be translated as well as the number of bases to be transcribed in the attenuator are large, hyper-sensitivity in the rate of gene expression to variation in the rate of amino acid synthesis emerges and can be combined with a high probability that initiation of transcription of the leader continues into the structural genes of the operon (see Figure 2). The importance of a large number of regulatory codons for sensitivity has been verified experimentally [39]. When, in contrast, there is competition between two single-step processes, the control is hyperbolic and lacks hyper-sensitivity [19]. Although competition between a single- and a multi-step process (exponential control) can lead to hyper-sensitivity, it is only at the cost of a very low probability for an initiation event to result in gene expression (see Figure 2; [19]).

The second source of hyper-sensitivity relates to the frequency *(f)* of occurrence of regulatory codons in the mRNAs of all translating ribosomes of the cell, just at the onset of amino acid starvation. A fractional decrease of amino acid synthesis is amplified by a factor of *1/f* in the resulting fractional increase in the time to translate regulatory codons (see Figure 3). This effect is a direct consequence of the stoichiometric coupling that exists between the rates of deacylation in protein synthesis of all the cell's tRNAs [16,22]. When amino acid limitation becomes increasingly severe, an ever-larger fraction of all ribosomes will expose the starved regulatory codons in their A site, until *f* becomes one and this sensitivity amplification effect vanishes.

When the regulatory codons in an attenuator are read by a tRNA that belongs to a family of isoaccepting tRNAs, cognate to the same amino acid, then hyper-sensitivity requires that the concentration of the aminoacylated form of the regulatory codon reader is sensitive to amino acid deprivation [16]. In short, the condition is that the concentrations of other members of the isoacceptor family are large in relation to the usage of their codons on all translating ribosomes, as compared to the concentration of the regulatory codon-reading tRNA in relation to the general usage of those codons in translation. When this condition is met, the charged level of the regulatory codon reader is maximally sensitive to amino acid limitation, while the charged levels of the other isoacceptors are insensitive and remain high ([16]; see Figure 4). In this case, the frequency *f* (Equation 4) refers to how often the regulatory and not all synonymous codons occur on translating ribosomes, which enhances the sensitivity at the onset of starvation. This is in line with results from experiments by Carter et al. [40], in which the three CUA codons, normally used in the *S.* *typhimurium* *leu* attenuator, were replaced by CUG codons. This led to reduced sensitivity, with total de-repression of the operon occurring only at severe Leu starvation. Our theory suggests that, for *E. coli,* CUA is the most suitable *leu* attenuator control codon, and that CUG is a less optimal but still a reasonable alternative [16]. On the assumption that the charged levels of Leu isoacceptors in *E. coli* and *S.* *typhimurium* react similarly to Leu limitation, our theory also predicts that if the CUA codons had been replaced by UUG or UUA codons, then the attenuator would have become even less sensitive to Leu limitation and the operon would not have been de-repressed even under severe Leu starvation.

Our modeling of the *his* and *trp* attenuators from *S.* *typhimurium* and *E. coli,* respectively, has reproduced available experimental data on these control circuits with respect to, in particular, regulatory range and basal expression levels. Furthermore, our simulations suggest that attenuation is close to fully relieved when the rate of Trp supply is about 70% of ribosomal demand, i.e., when *s* = 0.7 (Figure 6D). An *s* value of 0.7 roughly corresponds to a 30% reduction in growth rate due to Trp starvation [8,16,22], and our result can therefore be compared to experimental data from Yanofsky and Horn [38]. These show a 4-fold, out of a maximally 6-fold, relief of attenuation of the *trp* operon when the growth rate is reduced by 20% (*s* = 0.8) due to Trp limitation. These experimental data [38] are, therefore, in good agreement with our predictions (Figure 6D). For *s* values like these that are significantly less than one, the Trp as well as the Trp-tRNA^Trp^ levels are expected to be very low compared to their values at adequate supply of Trp [8,16,22].

Our analysis has also led to a novel suggestion regarding the role of the extra hair pin structure that exists in the* his*-attenuator of *S.* *typhimurium.* Our model predicts that removal of this hair pin results in a much higher basal level expression from the operon, which suggests that the hair pin serves to lower the probability of anti-terminator formation caused by translation termination and ribosome release from the attenuator. Accordingly, the presence of the extra hair pin leads to an increase of the regulatory range of the *his*-attenuator and reduces the metabolic cost associated with redundant His-synthesis when there is His in the medium.

Our analysis has, finally, resolved the “Ingraham-Maaløe-Neidhardt paradox” which states that the controlling ability of attenuation mechanisms necessarily leads to reduced growth rate [9], by showing that attenuators can indeed be hyper-sensitive regulators of amino acid synthesis. We also suggest that further improvement of attenuator performance comes from burst-like expression from attenuator-controlled amino acid biosynthetic operons, so that total protein elongation in the cell is marginally limited by amino acid supply only a small fraction of the time. This hypothesis is now addressed experimentally (J. Elf, in preparation).

## Supporting Information

### Selective Charging

To estimate the effect of selective charging, we must explicitly introduce concentrations of aminoacylated tRNA isoacceptors. From this, the rate of reading a specific codon is given by


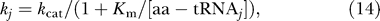


where *k*_cat_ and* K*_m_ are the Michaelis-Menten parameters for peptide elongation. The concentration of cognate aminoacylated tRNA that can read the codon is [aa-tRNA*_j_*]. The average time it takes to read the codon is *τ_j_ =* 1*/k_j_*. With these definitions, the average rate of protein synthesis, *v,* is given by [16,41]:


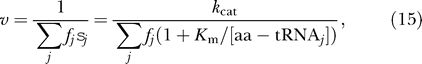


where *f*_j_ is the codon usage frequency of codon *j*.

When one amino acid is rate-limiting for protein synthesis, the tRNAs for the other amino acids are fully charged [22]. Assume, as in the main text, that the limiting amino acid has two cognate codons, c_a_ and c_b_. Codon c_a_ can be read by tRNA isoacceptor *A* with concentration *[A],* and c_b_ can be read by isoacceptor *B* with concentration *[B],* respectively. The charged fractions of *A* and *B* are *α* and *β,* respectively. In this case, Equation 15 becomes


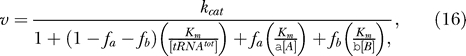


where *K_m_ =* 1*μM* and a total concentration of tRNA that can read an individual codon *[tRNA^tot^]* = 3.33 μM. Further, we chose *k*_cat_= 26 *s* ^−1^ in order to make *k_max_*= 20*s* ^−1^ at full charging, which facilitates comparison with Figure 3.

The rates of aminoacylation of the two isoacceptors are assumed to be proportional to the concentrations of their deacylated forms, i.e., to (1 − *α*)*[A]* and (1 − *β*)*[B].* If this were not the case, the computations would have to be modified but the same principles would pertain [16]. At the steady state, the rate of aminoacylation equals the rate of consumption of aminoacylation tRNA in protein synthesis, which is the frequency of the codons the isoacceptors reads, *f_a_* or *f_b_,* respectively, multiplied by the concentration of elongating ribosomes, [R], multiplied by the average rate of peptide elongation, *v*. This gives the flow balance relations


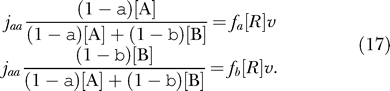


Equation 17 defines the charged fractions *α* and *β* for each amino acid supply level *s* = *v*/*v*_max_ *,* as given by variation in *j_aa_*. The rate of translation of the attenuation control codon is given by *k_j_ = k_cat_*/(1 + *K_m_*/(*α[A]*)), where *[A]* = 3.33 μM. The curves in Figure 4 are given for *[B]* = 3.33 μM, *[B]* = 1.67 μM, and *[B]* = 6.67 μM**,** respectively, while *f_a_* = *f_b_* = 0.025 for all curves.

## Abbreviations

nt: nucleotide
RNAP: RNA polymerase
